# Dose-dependent tuning of HSP70–Beclin-1 by kaempferol governs autophagy and chemosensitivity

**DOI:** 10.3389/fonc.2026.1802609

**Published:** 2026-05-20

**Authors:** Suqin Hu, Yanan Guo, Mingyan Li, Fangli Jin, Lei Luo, Zhihuan Yan, Jing Wang, Bao Liu, Qinsheng Zhang, Wenzhao Luo

**Affiliations:** 1 Department of Hepatobiliary Spleen and Stomach, Henan Province Hospital of Traditional Chinese Medicine, Zhengzhou, China; 2 Second Clinical Medical College, Henan University of Traditional Chinese Medicine, Zhengzhou, China; 3 Department of Respiratory and Critical Care Medicine, Henan Provincial People’s Hospital, Zhengzhou, China

**Keywords:** AMPK/mTOR signaling, autophagy directionality, dose-dependent regulation, HSP70–Beclin-1 complex, kaempferol

## Abstract

**Introduction:**

Autophagy can either support cell survival or promote cell death in response to therapeutic stress, yet the molecular switch that determines this directionality remains poorly defined.

**Methods:**

Using kaempferol as a proof‑of‑concept flavonoid and esophageal squamous carcinoma cells as a model system, we investigated its dose‑dependent effects on the HSP70–Beclin‑1 complex. *In vivo* experiments were also performed to evaluate kaempferol’s modulation of cisplatin’s antitumor activity.

**Results:**

Low‑dose kaempferol stabilized the HSP70–Beclin‑1 association, suppressed autophagic flux, and attenuated chemotherapy‑induced apoptosis. In contrast, high‑dose kaempferol disrupted this complex and enhanced autophagic flux through coordinated activation of AMPK/mTOR inhibition and ER stress–JNK signaling, leading to robust autophagy‑dependent chemosensitization. In vivo, high‑dose kaempferol potentiated cisplatin’s antitumor activity, whereas low‑dose kaempferol diminished it.

**Discussion:**

These findings reveal a generalizable molecular logic in which dose‑dependent modulation of a chaperone–autophagy initiation complex governs autophagy directionality and therapeutic response. By elucidating how a single compound can toggle this central regulatory node, the study provides a conceptual framework for optimizing the dosing of natural products and for leveraging autophagy‑based chemosensitization strategies across diverse tumor contexts.

## 1 Introduction

Autophagy is a fundamental stress-response pathway that enables cells to maintain homeostasis and adapt to metabolic or therapeutic pressure. In cancer, however, autophagy exhibits paradoxical, context-dependent roles: it may sustain tumor cell survival under mild stress yet facilitate cell death when excessively activated (1, 2). Although autophagy has been widely implicated in chemoresistance, the molecular determinants that govern whether autophagy acts as a cytoprotective or cytotoxic process remain poorly defined (3). Identifying regulatory nodes that dictate the *directionality* of autophagy is therefore essential for harnessing this pathway for therapeutic benefit.

The HSP70–Beclin-1 complex represents a critical point of convergence between chaperone-mediated proteostasis and autophagy initiation (4). Stabilization of this complex suppresses autophagy activation, whereas its dissociation permits Beclin-1–driven autophagy initiation (5). Despite its central position, it is unknown whether pharmacological modulation of this complex can operate as a tunable switch that controls autophagy outcomes, and whether such regulation follows a dose-dependent logic.

Kaempferol, a dietary flavonoid abundant in fruits and medicinal plants, is reported to exert broad biological effects (6, 7). Like many natural products, kaempferol exhibits dose-dependent paradoxical actions—cytoprotective at low levels but pro-stress and pro-apoptotic at higher levels—yet the molecular logic underlying this biphasic behavior remains obscure (8, 9). Moreover, whether such dose-dependent effects interface with autophagy regulation and stress signaling networks to influence therapeutic responses is unexplored (10).

In this study, using esophageal squamous carcinoma cells as a model system, we uncover a generalizable dose-dependent molecular switch in which kaempferol differentially modulates the HSP70–Beclin-1 complex to determine the direction of autophagic flux. Low-dose kaempferol strengthens HSP70–Beclin-1 binding and suppresses autophagy, whereas high-dose kaempferol disrupts this complex and integrates AMPK activation with ER stress–JNK signaling to markedly enhance autophagy. This mechanism forms the basis for a bidirectional effect on apoptosis and chemosensitivity. Our findings reveal a tunable, dose-governed regulatory architecture that may extend beyond kaempferol, offering conceptual and translational implications for leveraging natural compounds to modulate autophagy across diverse cancer contexts.

## 2 Materials and methods

### 2.1 Cell culture and drug treatment

KYSE150, KYSE450, HCT116, HepG2, HeLa, and A549 cells were purchased from MeisenCTCC (China). Cells were maintained at 37 °C in a humidified atmosphere with 5% CO_2_. KYSE150 and KYSE450 cells were cultured in RPMI-1640 medium (Gibco, USA), whereas the remaining cell lines were maintained in high-glucose DMEM (Gibco, USA). All media were supplemented with 10% fetal bovine serum (Gibco, USA) and 1% penicillin–streptomycin (HyClone, USA).

Kaempferol (Sigma-Aldrich, USA) was dissolved in DMSO to prepare a 100 mM stock solution and diluted to final concentrations of 0, 1, 2.5, 5, 10, 20, and 40 μM in culture medium (final DMSO concentration ≤0.1%). The 2.5 μM and 20 μM concentrations were defined as low-dose (Kaemp-L) and high-dose (Kaemp-H), respectively. Cisplatin (MedChemExpress, USA) was freshly prepared in sterile water before use and applied for 24–48 h.

For combination treatments, cells were pretreated with kaempferol for 2 h prior to cisplatin exposure. Inhibitors were added 1 h before kaempferol for mechanistic studies: 3-MA (5 mM, Sigma-Aldrich), Compound C (10 μM, MedChemExpress), 4-PBA (2 mM, Sigma-Aldrich), and SP600125 (10 μM, MedChemExpress). DMSO-treated cells served as solvent controls.

### 2.2 Cell viability assay (CCK-8)

Cells (3–5 × 10³ per well) were seeded in 96-well plates and treated with graded concentrations of kaempferol for 48 h. CCK-8 reagent (10 μL; Dojindo, Japan) was added, and absorbance at 450 nm was measured after 1–2 h of incubation at 37 °C using a microplate reader (BioTek, USA).

For cisplatin sensitivity assays, cells were treated with cisplatin alone or in combination with kaempferol for 48 h. IC_50_ values were calculated using nonlinear regression in GraphPad Prism (GraphPad Software, USA). Drug interactions were evaluated using CompuSyn (ComboSyn Inc., USA) to determine the Combination Index (CI), with CI < 1 indicating synergism.

### 2.3 Apoptosis analysis by flow cytometry

Following treatment, cells were harvested with trypsin lacking EDTA, washed with PBS, and stained using the Annexin V–FITC/PI apoptosis kit (Beyotime, China). Samples were analyzed on a BD FACSCanto II flow cytometer (BD Biosciences, USA). Data were processed using FlowJo software. Total apoptosis rates were calculated as the sum of early (Annexin V^+^/PI^-^) and late (Annexin V^+^/PI^+^) apoptotic cells.

### 2.4 Caspase-3 and Caspase-9 activity assays

Cells were lysed in Beyotime lysis buffer and incubated on ice for 15–30 min. Lysates were centrifuged at 12,000 × g, and protein concentrations were quantified using the BCA assay (Thermo Fisher Scientific, USA). Caspase-3 and Caspase-9 activities were measured following incubation of equal protein amounts with Ac-DEVD-pNA or Ac-LEHD-pNA substrates (Beyotime, China) at 37 °C for 1–2 h. Absorbance at 405 nm was recorded, and activity levels were normalized to protein content.

### 2.5 Western blot analysis

Cells were lysed in RIPA buffer (Beyotime, China) and centrifuged after 30 min on ice. Protein concentrations were determined via BCA assay. Equal protein samples were separated by SDS-PAGE and transferred onto PVDF membranes (Millipore, USA). Membranes were blocked with 5% BSA and incubated overnight at 4 °C with primary antibodies (**Table 1**). HRP-conjugated secondary antibodies (Cell Signaling Technology, USA) were applied for 1 h at room temperature. Signals were developed using ECL reagents (Thermo Fisher Scientific) and acquired with a Bio-Rad imaging system. Band intensities were quantified using ImageJ, normalized to β-actin or GAPDH.

**Table 1:** Antibody information.

Antibody	Concentration	Number	Company
Anti-beta Actin antibody	1/1000	ab8226	Abcam, England
Anti-Hsp70 antibody [5A5]	1/1000	ab2787	Abcam, England
Anti-Caspase-3 antibody [EPR18297]	1/2000	ab184787	Abcam, England
Anti-Cleaved Caspase-3 antibody [E83-77]	1/500	ab32042	Abcam, England
Anti-Caspase-9 antibody [EPR18107]	1/2000	ab202068	Abcam, England
Cleaved Caspase-9 (Asp315) (D8I9E) Rabbit Monoclonal Antibody	1/100	Asp315	Cell Signaling Technology, USA
Anti-PARP1 antibody [EPR18461]	1/1000	ab191217	Abcam, England
Anti-Cleaved PARP1 antibody [E51]	1/5000	ab32064	Abcam, England
Anti-Bcl-2 antibody [EPR17509]	1/2000	ab182858	Abcam, England
Anti-Bax antibody [E63]	1/3000	ab32503	Abcam, England
Anti-LC3B antibody [EPR18709]	1/2000	ab192890	Abcam, England
Anti-SQSTM1 / p62 antibody [EPR18351]	1/1000	ab207305	Abcam, England
Anti-Beclin 1 antibody [EPR19662]	1/2000	ab207612	Abcam, England
Anti-APG5L/ATG5 antibody [EPR1755(2)]	1/6000	ab108327	Abcam, England
Anti-LAMP1 antibody [EPR21026]	1/1000	ab208943	Abcam, England
Anti-AMPK alpha 1 antibody [Y365]	1/2500	ab32047	Abcam, England
Anti-AMPK alpha 1 (phospho T183) + AMPK alpha 2 (phospho T172) antibody [EPR5683]	1/1000	ab133448	Abcam, England
Anti-mTOR antibody [Y391]	1/3000	ab32028	Abcam, England
Anti-mTOR (phospho S2448) antibody [EPR426(2)]	1/5000	ab109268	Abcam, England
Anti-CHOP antibody [9C8]	1/1000	ab11419	Abcam, England
Anti-JNK1 + JNK2 + JNK3 antibody [EPR16797-211]	1/1000	ab179461	Abcam, England
Anti-JNK1 (phospho Y185) + JNK2 (phospho Y185) + JNK3 (phospho Y223) antibody [EP1597Y]	1/5000	ab76572	Abcam, England
Goat Anti-Rabbit IgG H&L	1:10000	ab150077	Abcam, England
Goat Anti-Mouse IgG H&L	1:10000	ab150113	Abcam, England

### 2.6 Quantitative real-time PCR

Total RNA was extracted using TRIzol (Invitrogen, USA). cDNA was synthesized using a reverse transcription kit (Takara, Japan). qPCR was performed with SYBR Green Master Mix (Takara, Japan) on an ABI 7500 Fast system (Applied Biosystems, USA). Gene expression levels were calculated using the 2^-ΔΔCt method with β-actin as the internal control. Primer sequences are listed in **Table 2**.

**Table 2:** Primer sequences.

Gene	NM number	Forward primer	Reverse primer
HSP70	XM-054352493.1	TGTTGATAAGTTGGCTGAA	ATACTGGTCCTCCTTGTT
β-actin	NM-001101.5	CTCTTCCAGCCTTCCTTCCT	AGCACTGTGTTGGCGTACAG

### 2.7 Immunofluorescence staining

Cells grown on coverslips were fixed in 4% paraformaldehyde (Solarbio), permeabilized with 0.1% Triton X-100, and blocked with 5% BSA. Cells were incubated with primary antibodies against HSP70 at 4 °C overnight, followed by fluorophore-conjugated secondary antibodies (Invitrogen, USA). Nuclei were stained with DAPI (Beyotime, China). Images were captured using a Leica SP8 confocal microscope with identical exposure settings across samples.

### 2.8 Autophagic flux assay

Cells were transfected with the mRFP-GFP-LC3 reporter (Beyotime) for 24–48 h before drug treatment. After washing and replacing with non-fluorescent medium, images were obtained using a Leica SP8 confocal microscope. Yellow (GFP^+^/RFP^+^) puncta indicated autophagosomes, whereas red-only puncta represented autolysosomes. Autophagic flux was quantified as the ratio of red to yellow puncta.

### 2.9 Co-immunoprecipitation

Cells were lysed in IP lysis buffer (Beyotime). Lysates were precleared with Protein A/G magnetic beads (Thermo Fisher Scientific). Equal protein amounts were incubated with Beclin-1 antibody and magnetic beads overnight at 4 °C. Immunoprecipitates were washed and eluted by boiling in SDS loading buffer. HSP70–Beclin-1 interactions were analyzed by Western blot.

### 2.10 HSP70 knockdown by shRNA

shRNA sequences targeting HSP70 were cloned into lentiviral vectors (GeneChem, China). Viral supernatants were used to infect KYSE150 and KYSE450 cells in the presence of polybrene. Stable lines were selected using puromycin (Sigma-Aldrich) and validated by Western blot and qPCR.

### 2.11 Xenograft mouse model

All animal procedures were approved by the Animal Ethics Committee of Henan Provincial Hospital of Traditional Chinese Medicine (AWEC -2505-014). BALB/c nude mice (6 weeks old) were maintained under SPF conditions with free access to food and water.

KYSE150 cells (5 × 10^6^) were injected subcutaneously into the right axilla. Mice were randomized into four groups: Control, CDDP, Kaemp-L + CDDP (2.5 mg/kg), and Kaemp-H + CDDP (20 mg/kg). Kaempferol was administered daily (intraperitoneally or gavage), and cisplatin (3 mg/kg) weekly by intraperitoneal injection.

Tumor length (L) and width (W) were measured every 2–3 days, and volume was calculated as 0.5 × L × W². Tumors were excised, measured, and weighed at study endpoint.

### 2.12 Immunohistochemistry

Tumors were fixed in 10% neutral buffered formalin, embedded in paraffin, and sectioned at 4 μm. After deparaffinization, antigen retrieval, and blocking, sections were incubated with primary antibodies, followed by HRP-conjugated secondary antibodies and DAB development. Hematoxylin counterstaining was performed. Staining intensity and positivity were quantified using the H-score method.

### 2.13 Statistical analysis

Data are presented as mean ± SD. Two-group comparisons used two-tailed unpaired *t*-tests. Multi-group analyses used one-way ANOVA with Bonferroni *post hoc* correction. IC_50_ values were calculated via nonlinear regression. Drug synergy was assessed using the Combination Index (CI). Statistical significance was defined as *P* < 0.05.

## 3 Results

### 3.1 Dose-dependent effects of kaempferol on cell viability, apoptosis, and cisplatin sensitivity

**Figure 1:**
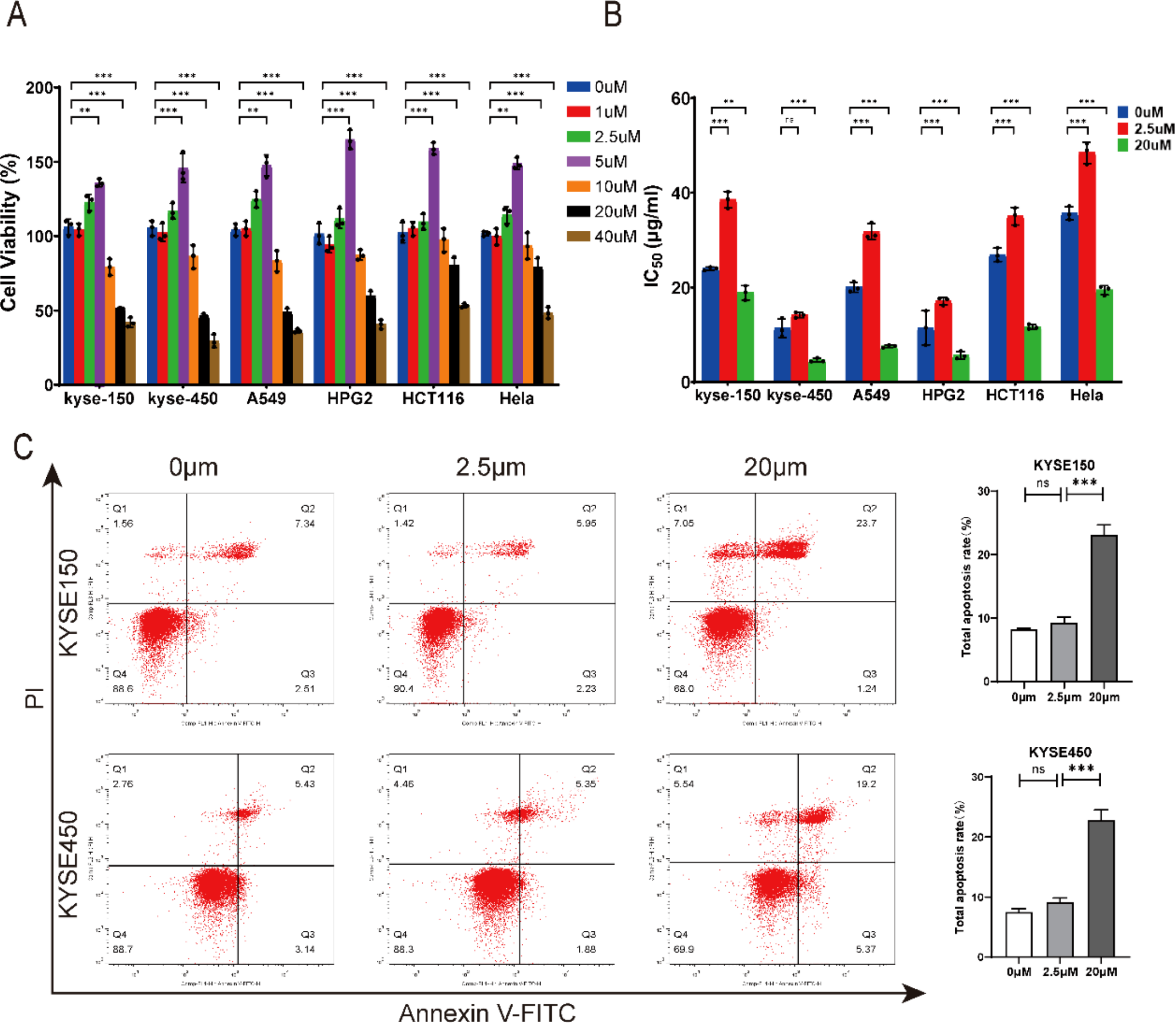
Effects of different concentrations of kaempferol on chemosensitivity of tumor cells. **(A)** CCK-8 assay showing the impact of varying concentrations of kaempferol on different tumor cell lines. **(B)** Influence of kaempferol concentration on the IC_50_ of cisplatin in different tumor cell lines. **(C)** Flow cytometry analysis of apoptosis in KYSE150 and KYSE450 cells treated with different concentrations of kaempferol. ns: no significant difference, **p < 0.01, ***p < 0.001.

To investigate the biological effects of kaempferol, we first examined its dose-dependent impact on cell viability across multiple cancer cell lines. CCK-8 assays showed that kaempferol at 1–5 μM slightly increased viability in KYSE150, KYSE450, A549, HepG2, HCT116, and HeLa cells, whereas higher concentrations (10–40 μM) induced a marked, dose-dependent reduction in viability (**Figure 1A**). These results reveal a clear biphasic regulatory pattern.

We next assessed whether kaempferol alters cisplatin sensitivity. Treatment with 2.5 μM kaempferol increased the IC_50_ values for cisplatin in all cell lines, whereas 20 μM kaempferol significantly reduced IC_50_ values (**Figure 1B**), indicating that low-dose kaempferol attenuates cisplatin cytotoxicity, while high-dose kaempferol enhances chemosensitivity. Flow cytometry further supported this trend: 20 μM kaempferol markedly increased apoptosis in KYSE150 and KYSE450 cells (*P* < 0.001), whereas 2.5 μM had minimal effect (**Figure 1C**).

### 3.2 Low-dose kaempferol upregulates HSP70 expression and suppresses cisplatin-induced autophagy

**Figure 2:**
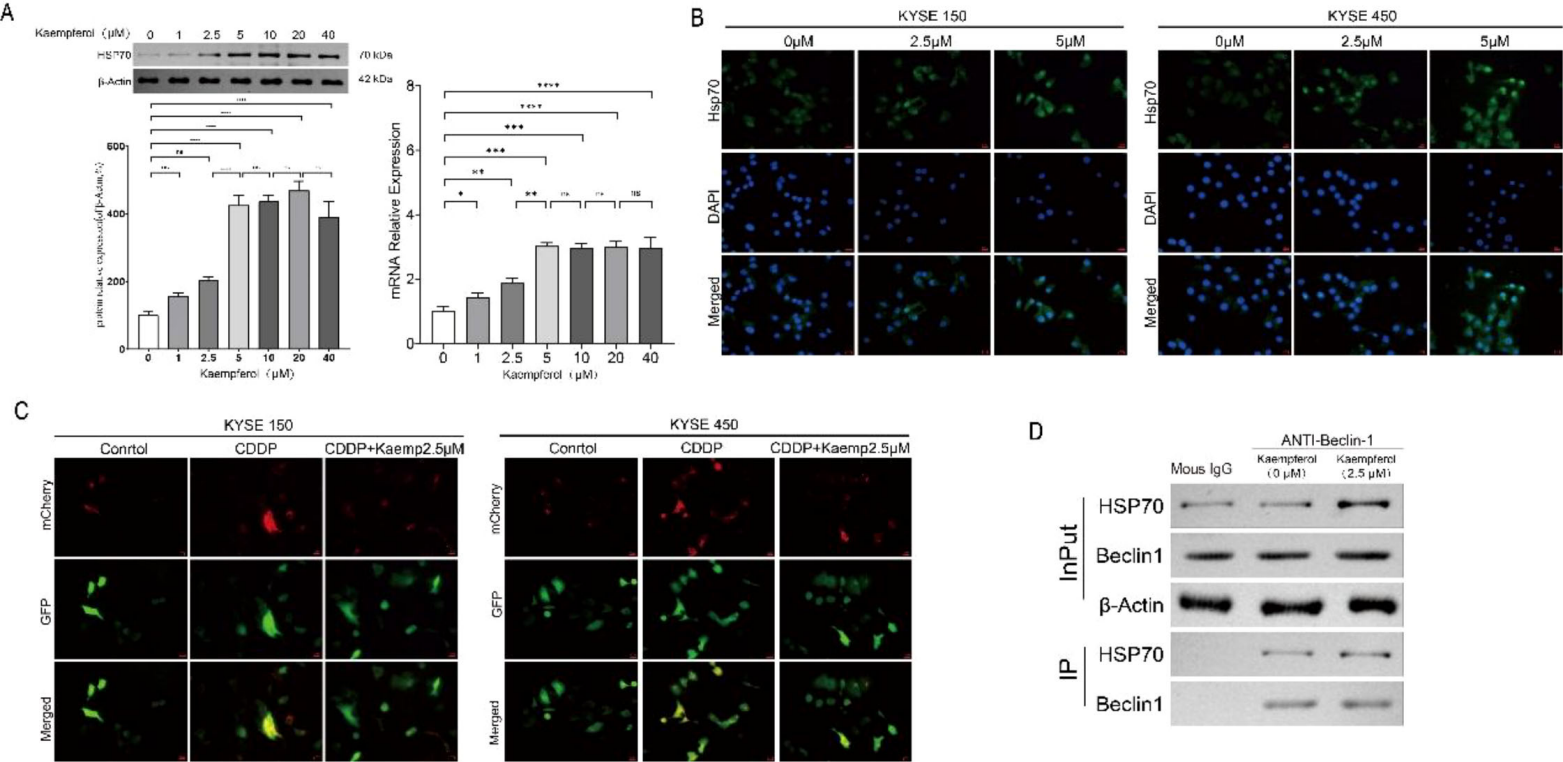
Low-dose kaempferol upregulates HSP70 expression in KYSE150 and KYSE450 cells. **(A)** qPCR and Western blot (WB) analysis of HSP70 expression after treatment with different doses of kaempferol. **(B)** Cellular immunofluorescence (IF) staining showing HSP70 expression in KYSE150 and KYSE450 cells. **(C)** Immunofluorescence analysis of autophagic activity in KYSE150 and KYSE450 cells treated with kaempferol (2.5 μM). **(D)** Co-immunoprecipitation (Co-IP) demonstrating that kaempferol enhances the HSP70–Beclin-1 interaction. *p < 0.05 compared to the two groups, **p < 0.01 compared to the two groups, ***p < 0.001 compared to the two groups, ****p < 0.0001 compared to the two groups.

To elucidate the cytoprotective mechanism of low-dose kaempferol, we examined its effect on HSP70 expression. Western blot analysis showed that 1–5 μM kaempferol induced a dose-dependent increase in HSP70 protein, while higher concentrations produced no further elevation (**Figure 2A**). qPCR confirmed parallel increases in HSP70 mRNA.

Immunofluorescence revealed that 2.5 and 5 μM kaempferol markedly increased HSP70 fluorescence intensity and induced partial perinuclear/nuclear redistribution (**Figure 2B**).

We next assessed the effect of low-dose kaempferol on cisplatin-induced autophagy. LC3 immunofluorescence indicated that cisplatin induced robust LC3 puncta formation, whereas 2.5 μM kaempferol attenuated this induction (**Figure 2C**).

Co-immunoprecipitation showed increased HSP70 binding to Beclin-1 following 2.5 μM kaempferol treatment, whereas total HSP70 levels remained unchanged (**Figure 2D**), indicating that low-dose kaempferol enhances HSP70–Beclin-1 complex formation, thereby restricting autophagy initiation.

### 3.3 The cytoprotective effect of low-dose kaempferol is dependent on HSP70

**Figure 3:**
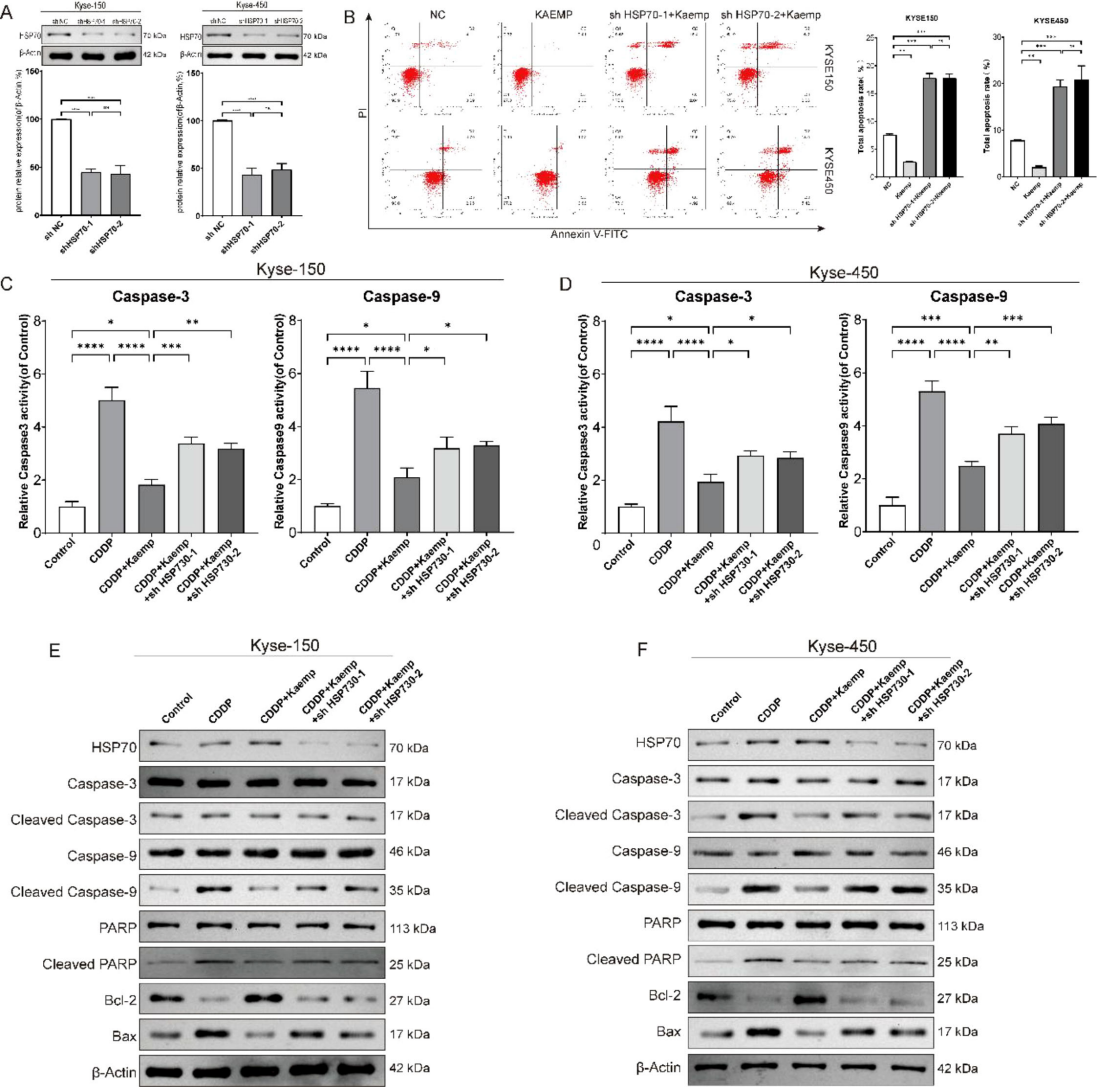
The anti-apoptotic mechanism of low-dose kaempferol depends on HSP70. **(A)** Western blot (WB) analysis confirming the successful establishment of HSP70-knockdown cell lines. **(B)** Flow cytometry analysis showing that the cytoprotective effect of low-dose kaempferol is attenuated after HSP70 knockdown. **(C, D)** Effects of low-dose kaempferol on Caspase-3 & 9 activity after HSP70 knockdown. **(E, F)** WB detection of apoptosis-related proteins: HSP70, Caspase-3/9, Cleaved Caspase-3/9, PARP, Cleaved PARP, Bcl2, and Bax. *p < 0.05 compared to the two groups, **p < 0.01 compared to the two groups, ***p < 0.001 compared to the two groups, ****p < 0.0001 compared to the two groups.

To determine whether HSP70 mediates the protective effects of low-dose kaempferol, HSP70-knockdown cell lines were generated. Western blotting confirmed efficient reduction of HSP70 expression (**Figure 3A**).

Low-dose kaempferol reduced cisplatin-induced apoptosis; however, this effect was abolished in HSP70-silenced cells (**Figure 3B**). Caspase-3 and Caspase-9 activation induced by cisplatin was suppressed by kaempferol but restored upon HSP70 knockdown (**Figures 3C, D**).

Consistently, Western blotting showed that kaempferol reduced levels of cleaved Caspase-3, cleaved Caspase-9, and cleaved PARP, while increasing Bcl-2; these effects were diminished when HSP70 was silenced (**Figures 3E, F**).

Additional autophagic flux experiments were performed using the mRFP-GFP-LC3 reporter in shNC and shHSP70 cells treated with cisplatin in the presence or absence of low-dose kaempferol. The results demonstrated that low-dose kaempferol markedly suppressed cisplatin-induced autophagic flux in control cells, whereas this inhibitory effect was largely abolished following HSP70 knockdown (**Supplementary Figure 1**). Together, these results demonstrate that HSP70 is essential for the anti-apoptotic effect of low-dose kaempferol. Moreover, mRFP-GFP-LC3 analysis showed that the suppression of cisplatin-induced autophagic flux by low-dose kaempferol was largely abolished after HSP70 knockdown. These findings indicate that HSP70 is not only required for the cytoprotective phenotype of low-dose kaempferol, but also mediates its inhibitory effect on autophagy, further supporting the HSP70-dependent regulation of the Beclin-1-associated autophagy machinery.

### 3.4 High-dose kaempferol disrupts the HSP70–Beclin-1 complex and markedly enhances autophagic flux

**Figure 4:**
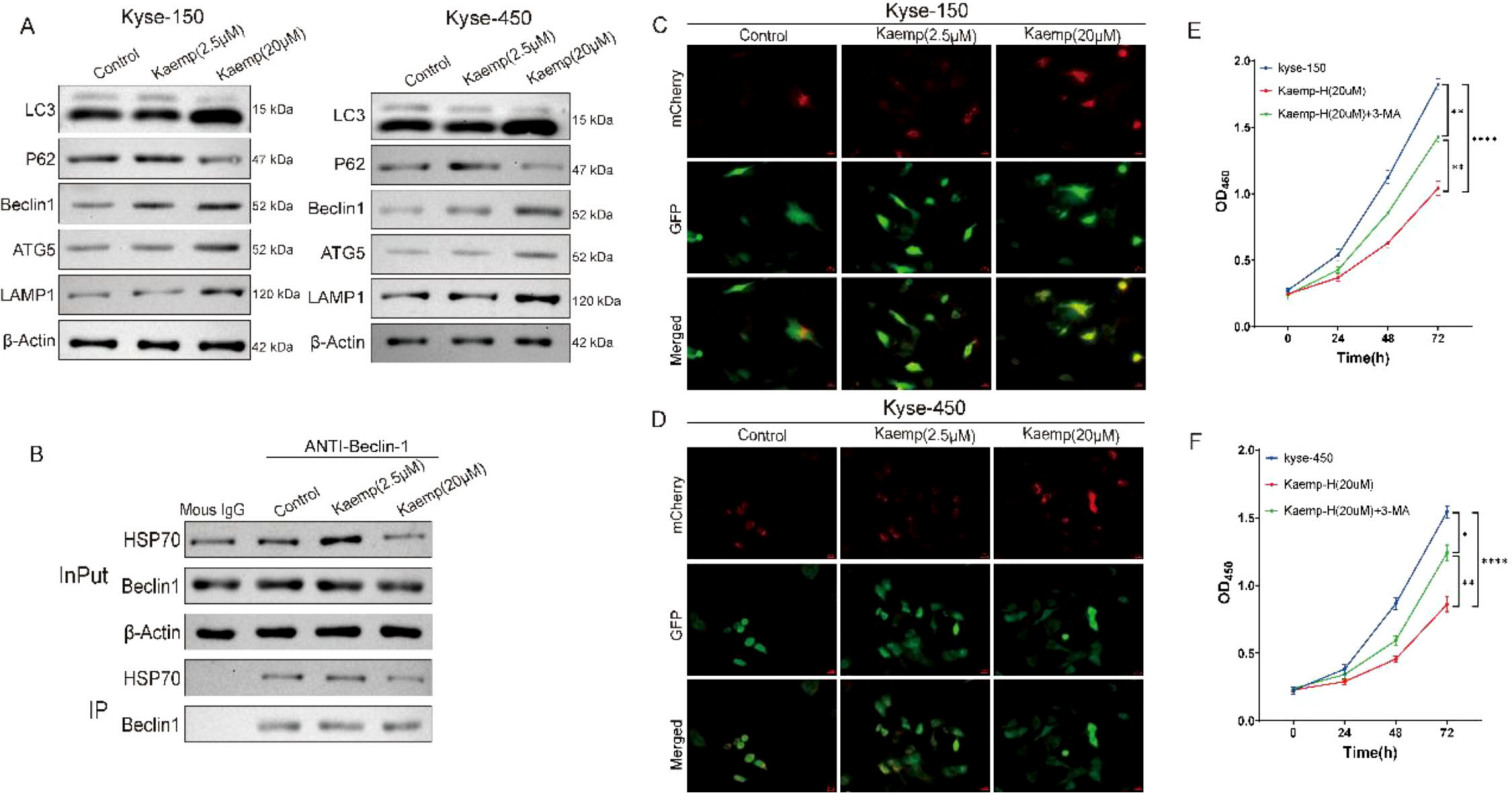
High-dose kaempferol disrupts the HSP70–Beclin-1 complex and enhances autophagosome formation. **(A)** Western blot (WB) analysis of the effects of high-dose kaempferol on the expression of autophagy markers: LC3-I/II, p62, Beclin-1, ATG5, and LAMP1. **(B)** Co-immunoprecipitation (Co-IP) assay showing that high-dose kaempferol reduces HSP70–Beclin-1 interaction. **(C, D)** Immunofluorescence analysis demonstrating that high-dose kaempferol increases autophagic flux (scale bar: 100 μm). **(E, F)** CCK-8 assay showing changes in cell viability after autophagy inhibition with 3-MA. *p < 0.05 compared to the two groups, **p < 0.01 compared to the two groups, ****p < 0.0001 compared to the two groups.

We next explored the mechanism underlying the effects of high-dose kaempferol. Western blotting revealed that 20 μM kaempferol significantly increased LC3-II, Beclin-1, ATG5, and LAMP1 levels, while decreasing p62 (**Figure 4A**). These findings indicate that high-dose kaempferol activates autophagy and promotes autophagic flux.

Co-IP analysis showed reduced HSP70 binding to Beclin-1 following 20 μM kaempferol treatment, without changes in input protein levels (**Figure 4B**), suggesting that high-dose kaempferol destabilizes the HSP70–Beclin-1 complex.

LC3 immunofluorescence further supported autophagy activation, showing robust LC3 puncta accumulation with 20 μM kaempferol (**Figures 4C, D**).

Functionally, 3-MA partially rescued the reduced viability induced by high-dose kaempferol (**Figures 4E, F**), demonstrating that enhanced autophagic flux contributes to its growth-inhibitory effect.

**Figure 5:**
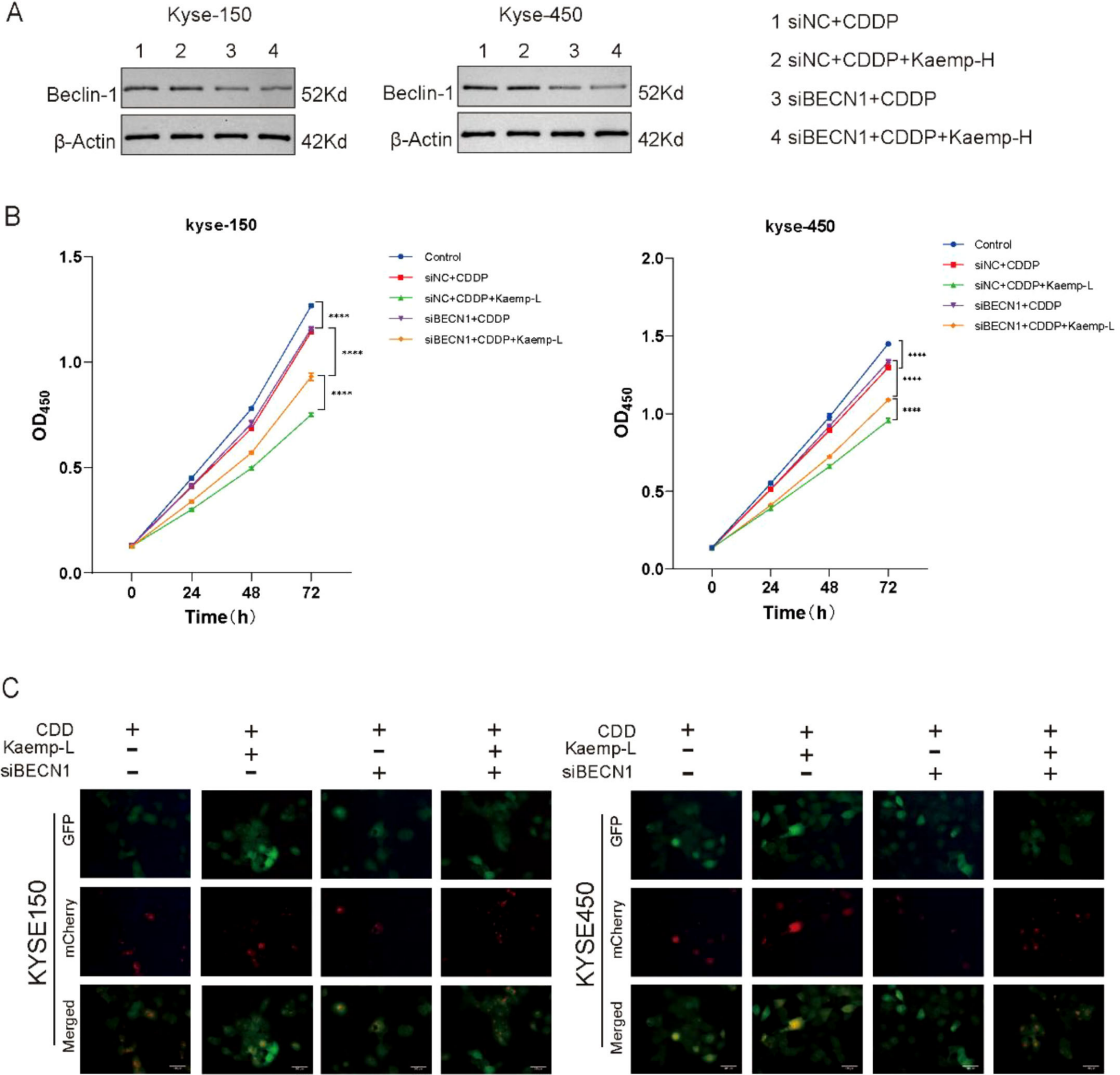
Effects of kaempferol on cell viability and autophagy following Beclin1 knockdown. **(A)** Western blot analysis confirming the knockdown efficiency of Beclin1 by siBeclin1. **(B)** Cell viability assessed by CCK-8 assay. Knockdown of Beclin1 significantly attenuated the additional reduction in cell viability induced by high-dose kaempferol. **(C)** Analysis of autophagic flux. Beclin1 knockdown markedly suppressed the increase in autophagic flux triggered by kaempferol. ****p < 0.0001.

Additionally, the effects of kaempferol on cell viability and autophagic flux were examined under Beclin1 knockdown conditions. The efficiency of Beclin1 silencing was confirmed by Western blotting (**Figure 5A**). Subsequently, cells were treated with cisplatin in the presence or absence of high-dose kaempferol. Cell viability was assessed, and autophagic flux was evaluated using the mRFP-GFP-LC3 reporter system. The results showed that, in control cells, high-dose kaempferol further enhanced cisplatin-induced suppression of cell viability and increased autophagic flux. In contrast, Beclin1 knockdown significantly attenuated the additional reduction in cell viability caused by high-dose kaempferol and markedly suppressed the increase in autophagic flux (**Figures 5B, C**). These findings suggest that the pro-death chemosensitizing effect of high-dose kaempferol is mediated, at least in part, through Beclin1−dependent autophagy.

### 3.5 High-dose kaempferol enhances cisplatin sensitivity through autophagy activation

**Figure 6:**
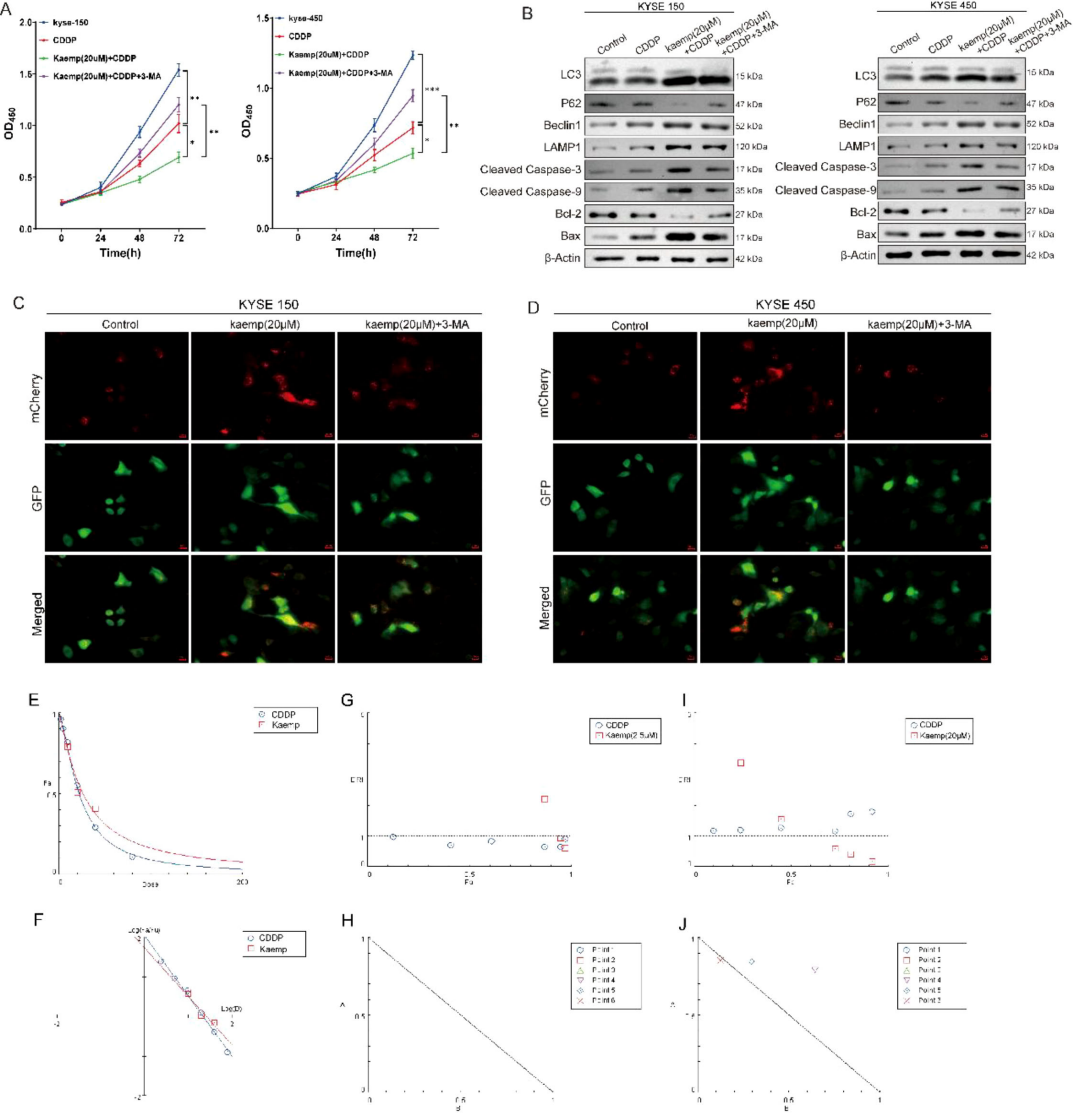
High-dose kaempferol induces autophagy in cancer cells and enhances chemosensitivity. **(A)** CCK-8 assay showing that 3-MA, an autophagy inhibitor, attenuates the enhanced chemosensitivity induced by high-dose kaempferol. **(B)** Representative Western blot images and grayscale analysis of autophagy- and apoptosis-related proteins. **(C, D)** Immunofluorescence analysis of the effects of 3-MA on autophagy. **(E–J)** CompuSyn analysis of the synergistic effects between different concentrations of kaempferol and cisplatin. *p < 0.05 compared to the two groups, **p < 0.01 compared to the two groups, ***p < 0.001 compared to the two groups.

Cisplatin significantly reduced viability in ESCC cells, and co-treatment with 20 μM kaempferol further suppressed viability compared with cisplatin alone (**Figure 6A**). This enhanced inhibition was attenuated by 3-MA, indicating autophagy dependence.

Western blot analysis showed increased LC3-II and Beclin-1, decreased p62, elevated LAMP1, and increased apoptotic markers (cleaved Caspase-3, cleaved Caspase-9, Bax), along with reduced Bcl-2 following combined treatment (**Figure 6B**). These changes were partially reversed by 3-MA.

LC3 immunofluorescence showed consistent results: 20 μM kaempferol markedly increased LC3 puncta, and its combination with cisplatin further enhanced LC3 accumulation, whereas 3-MA diminished these changes (**Figures 6C, D**).

CompuSyn analysis demonstrated synergistic cytotoxicity (CI < 1) between cisplatin and 20 μM kaempferol across multiple effect levels (**Figures 6E–J**). No synergy was observed with 2.5 μM kaempferol.

### 3.6 High-dose kaempferol regulates autophagy via AMPK/mTOR and ER stress–JNK pathways

**Figure 7:**
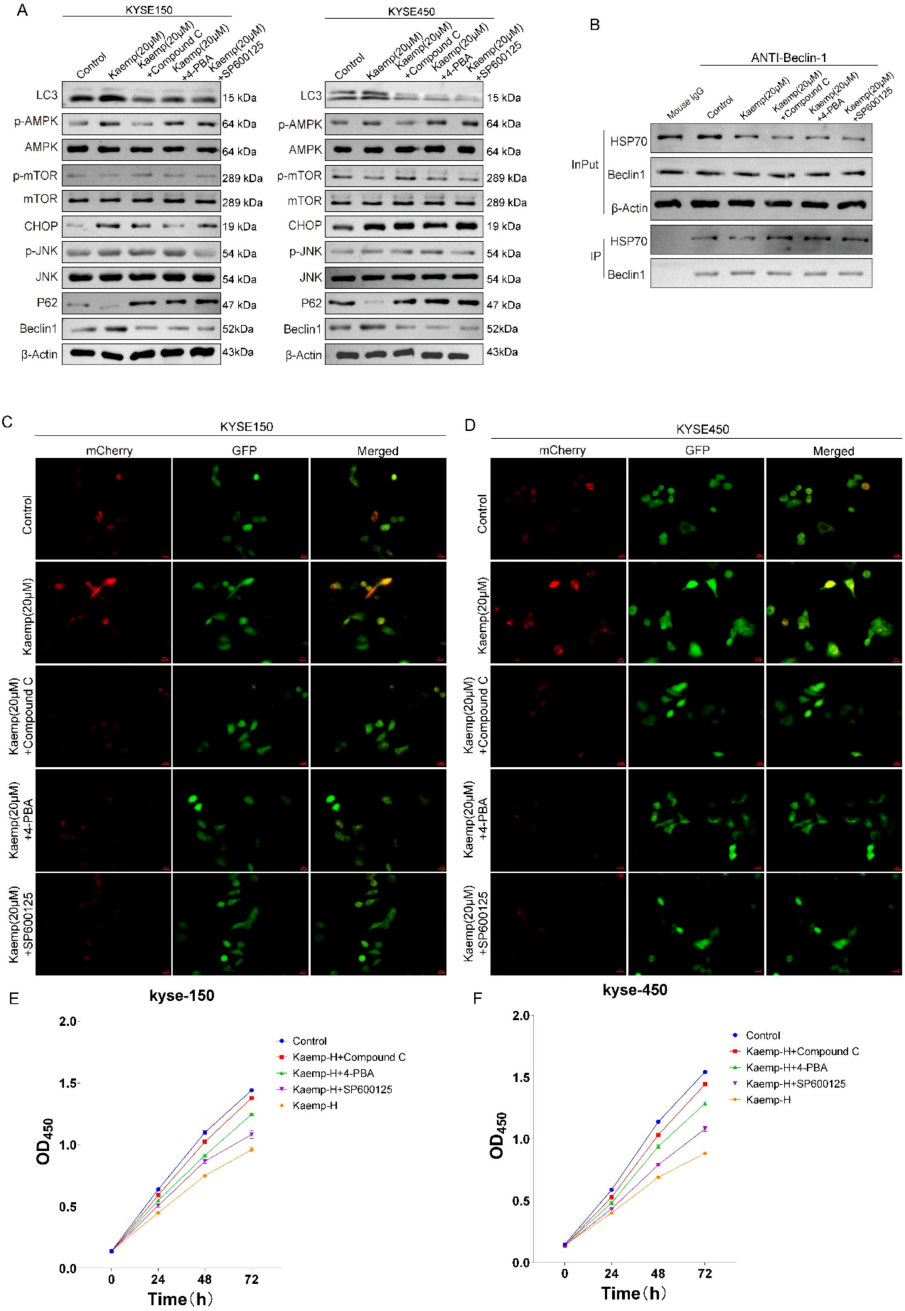
High-dose kaempferol disrupts the HSP70–Beclin-1 complex and enhances autophagic flux through activation of AMPK–mTOR and ER stress–JNK signaling pathways. **(A)** Western blot analysis of autophagy-related upstream pathway proteins. **(B)** Co-immunoprecipitation (Co-IP) assay showing that activation of related pathways disrupts the HSP70–Beclin-1 complex. **(C, D)** Autophagic flux detection demonstrating that high-dose kaempferol activates autophagy in a pathway-dependent manner. **(E, F)** Cell viability was assessed using the CCK-8 assay.

High-dose kaempferol activated multiple upstream pathways involved in autophagy regulation. Western blotting revealed increased p-AMPK and CHOP, decreased p-mTOR, and elevated p-JNK in both ESCC cell lines (**Figure 7A**). Inhibitors targeting AMPK (Compound C), ER stress (4-PBA), or JNK (SP600125) each reversed these effects and partially suppressed LC3-II accumulation and p62 degradation.

Co-IP analysis showed that inhibition of any of these pathways partially restored HSP70–Beclin-1 binding (**Figure 7B**), indicating that AMPK/mTOR, ER stress, and JNK pathways cooperatively mediate destabilization of the complex.

LC3 immunofluorescence confirmed that all three inhibitors reduced the LC3 puncta induced by high-dose kaempferol (**Figures 7C, D**).

To further determine whether these signaling pathways functionally contribute to the cytotoxic effect of high-dose kaempferol, we assessed cell viability in the presence of pathway inhibitors. CCK-8 assays showed that 20 μM kaempferol markedly reduced cell viability in ESCC cells, whereas co-treatment with Compound C, 4-PBA, or SP600125 each partially rescued this reduction (**Figures 7E, F**). These findings indicate that AMPK activation, ER stress, and JNK signaling are not only involved in the regulation of autophagic flux under high-dose kaempferol treatment, but also functionally contribute to its growth-inhibitory effect.

### 3.7 High-dose kaempferol enhances the antitumor activity of cisplatin *in vivo*

**Figure 8:**
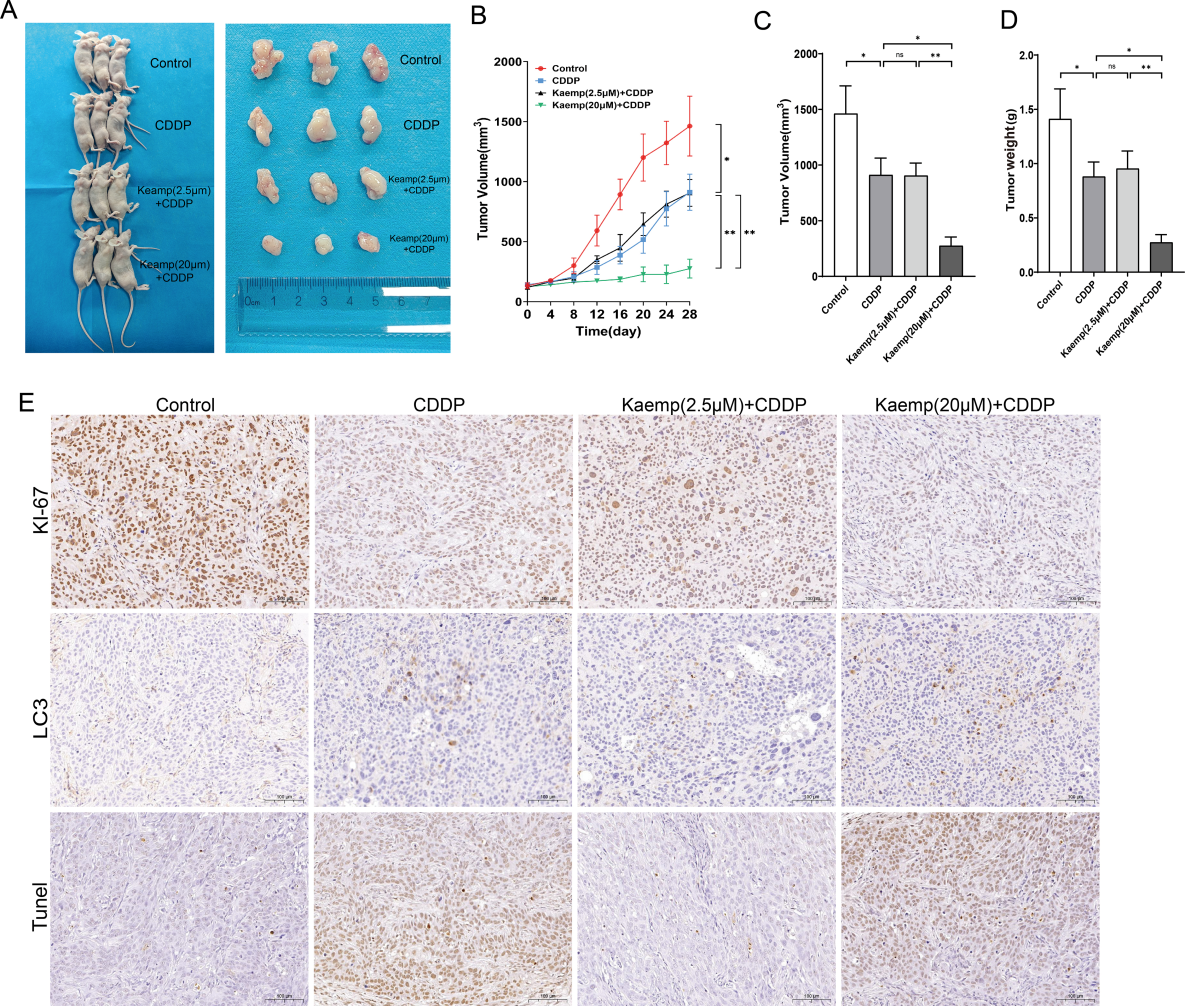
Xenograft tumor experiment in nude mice validates that high-dose kaempferol enhances tumor autophagy and chemosensitivity. **(A)** Representative images of nude mice and excised tumors. **(B)** Tumor growth curves. **(C)** Tumor volume measurements. **(D)** Tumor weight. **(E)** IHC analysis of Ki67, LC3, CHOP, and TUNEL expression in mouse tumor tissues. *p < 0.05 compared to the two groups, **p < 0.01 compared to the two groups.

In xenograft models, tumors formed successfully across all groups (**Figure 8A**). Cisplatin significantly reduced tumor growth, and kaempferol modulated this effect in a dose-dependent manner. Low-dose kaempferol attenuated cisplatin’s antitumor efficacy, increasing tumor volume and weight compared with cisplatin alone, whereas high-dose kaempferol further reduced tumor burden (**Figures 8B, C**).

Immunohistochemistry showed that high-dose kaempferol combined with cisplatin decreased Ki-67, enhanced LC3 staining, and increased TUNEL positivity (**Figure 8E**), indicating enhanced autophagy and apoptosis *in vivo*. Low-dose kaempferol exhibited the opposite trend.

## 4 Discussion

This study uncovers a generalizable molecular switch that governs the functional direction of autophagy through dose-dependent remodeling of the HSP70–Beclin-1 complex. Using kaempferol as a proof-of-concept molecule, we demonstrate that differential modulation of this complex determines whether autophagy acts in a cytoprotective or cytotoxic manner under chemotherapeutic stress. Although ESCC cells served as an experimentally tractable model system, the mechanisms described here involve conserved autophagy regulators and stress-signaling pathways, suggesting broader applicability across cancer types and potentially other stress-associated contexts.

Our findings integrate and extend several lines of prior research. Previous work has shown that HSP70 promotes tumor survival by stabilizing unfolded proteins, suppressing apoptosis, and limiting autophagy initiation (11–13). Studies have also revealed that Beclin-1 activity is tightly constrained by protein–protein interactions that regulate its release into active autophagy complexes (14). The present study connects these observations by demonstrating that HSP70–Beclin-1 association is not static but dynamically regulated by small molecules in a dose-dependent manner (12, 15). This provides mechanistic insight into how chaperone networks interface with autophagy under fluctuating stress levels.

Interestingly, the relationship between HSP70 and autophagy has been a subject of debate in the literature. Several studies have reported that HSP70 suppresses autophagy by stabilizing Beclin-1 or inhibiting the formation of the autophagy initiation complex (5, 16). In contrast, other investigations have demonstrated that HSP70 can promote autophagy under certain stress conditions, particularly when cells are exposed to proteotoxic or metabolic challenges (4, 12). Our findings offer a potential unifying framework to reconcile these seemingly contradictory observations. We demonstrate that the functional outcome of HSP70 modulation on autophagy is not fixed but rather depends on the intensity of the stress signal and the context of HSP70 regulation. Under mild stress (low-dose kaempferol), moderate HSP70 upregulation strengthens its interaction with Beclin-1, thereby restraining autophagy initiation. Under stronger stress (high-dose kaempferol), the coordinated activation of AMPK/mTOR and ER stress–JNK pathways overrides the HSP70–Beclin-1 association, leading to its dissociation and subsequent enhancement of autophagic flux. This dose-dependent, context-sensitive regulation may explain why HSP70 has been reported to both suppress and promote autophagy in different experimental settings, with the net effect likely determined by the cellular stress intensity, the degree of HSP70 induction, and the concurrent activation of stress signaling pathways that modulate the chaperone–autophagy interface.

The dual effects of kaempferol observed here are also consistent with reports that flavonoids exhibit dichotomous biological behaviors: lower concentrations often mitigate oxidative or metabolic stress, whereas higher concentrations induce ER stress, mitochondrial dysfunction, or apoptosis (17, 18). However, the underlying mechanisms have remained diffuse, lacking a unifying principle. Our results provide a conceptual framework that dose-dependent regulation of a nodal protein complex—rather than generalized “stress intensity”—acts as the decisive determinant that toggles autophagy between protective and pro-death outputs. This mechanistic model may also apply to other natural compounds reported to exert biphasic effects on autophagy, including quercetin and resveratrol, highlighting its potential generality.

Mechanistically, the data reveal that high-dose kaempferol activates AMPK and ER stress–JNK signaling while suppressing mTOR, and inhibitors of these pathways partially restore HSP70–Beclin-1 association. These findings support a distributed regulatory network in which multiple stress-responsive pathways converge on a shared autophagy-initiating node (19). Similar multilayered regulation has been described in studies examining autophagy induction by metabolic stress, proteotoxic stress, and nutrient deprivation, reinforcing the notion that autophagy directionality is governed by integrated signaling thresholds rather than single linear pathways (20, 21).

The *in vivo* findings further validate the model by showing that high-dose kaempferol amplifies chemotherapy-induced apoptosis, whereas low-dose kaempferol attenuates it, reflecting the bidirectional rewiring of stress responses at the molecular level (22, 23). Importantly, these results raise a translational consideration: natural products commonly consumed as health supplements may inadvertently confer chemoresistance when used at subtherapeutic doses, a point that warrants careful clinical evaluation (24).

Although the study provides a coherent mechanistic framework, several aspects warrant further exploration. The pharmacokinetic behavior of kaempferol at higher doses remains to be fully characterized, and advanced structural approaches will be needed to resolve the conformational dynamics of HSP70–Beclin-1 dissociation at atomic resolution. Moreover, kaempferol likely interfaces with additional processes—including oxidative stress modulation, mitochondrial fitness, and DNA repair—that may interact with autophagy in shaping therapeutic responses (25–27). These considerations reflect natural extensions of the current work rather than fundamental limitations and offer opportunities to refine the proposed model.

Together, these findings provide a mechanistic paradigm in which dose-dependent modulation of the HSP70–Beclin-1 complex determines autophagy directionality and therapeutic responsiveness. By revealing how a single compound can toggle this core decision node, the study establishes a framework for rational dose optimization of natural products and suggests broader opportunities to exploit autophagy-based chemosensitization strategies across diverse tumor settings.

## Data Availability

The original contributions presented in the study are included in the article/**Supplementary Material**. Further inquiries can be directed to the corresponding author/s.
