# Supplementary figures and images for: Dose-dependent tuning of HSP70–Beclin-1 by kaempferol governs autophagy and chemosensitivity
Source: Front Oncol. 2026 May 20;16:1802609. doi: 10.3389/fonc.2026.1802609 (PMC13230012; doi:10.3389/fonc.2026.1802609)

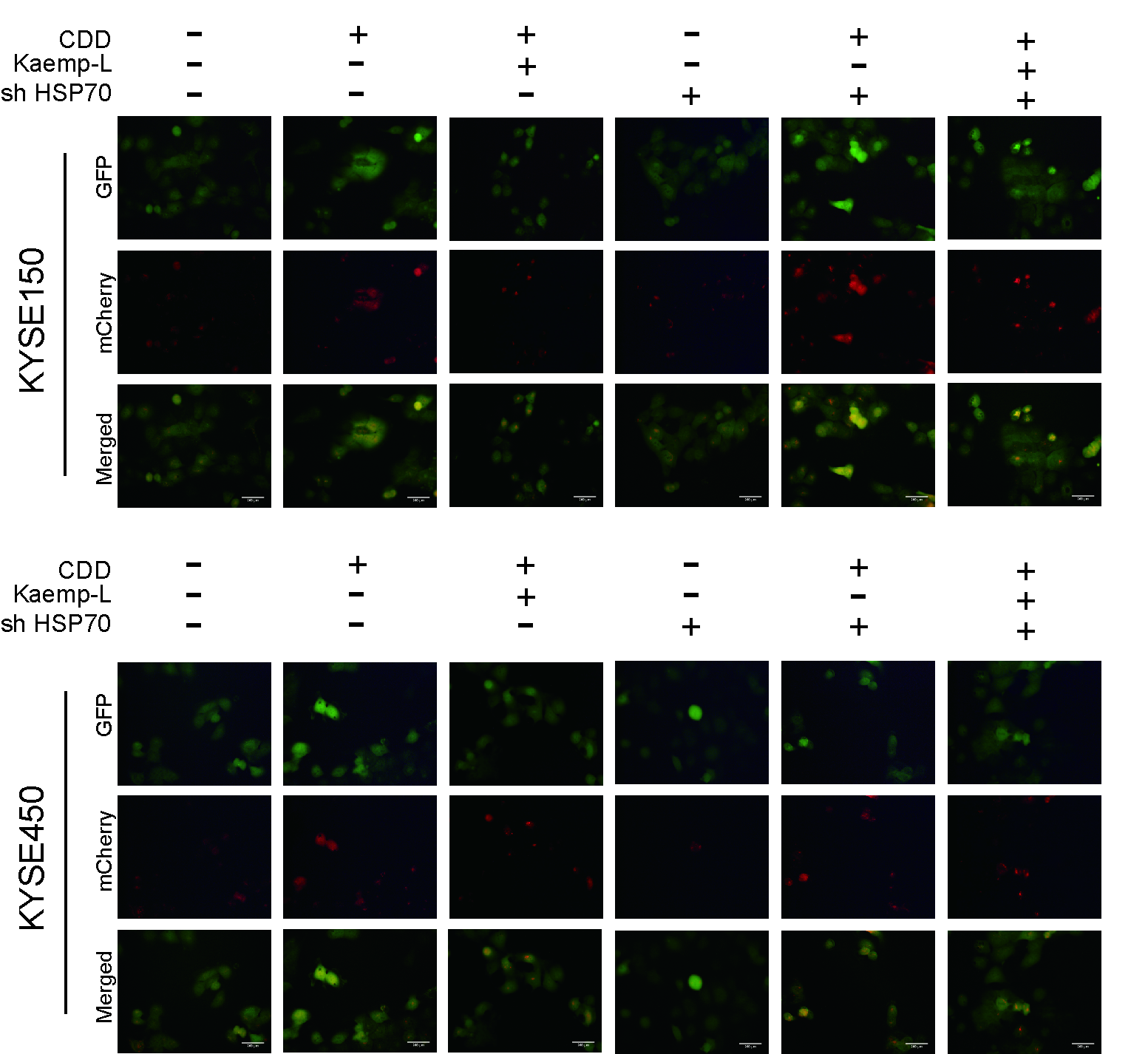

Supplement: Supplementary file 1 [file Image1.tif]
